# Does Early Decompressive Craniectomy Improve Outcome? Experience from an Active UK Recruiter Centre

**DOI:** 10.1155/2013/714945

**Published:** 2013-02-20

**Authors:** E. García Vicente, V. Garnelo Rey, M. Manikon, S. Ashworth, M. H. Wilson

**Affiliations:** ^1^Adult Intensive Care Unit, St. Mary's Hospital, Imperial College NHS Trust, London W2 6HP, UK; ^2^Neurosurgery Department, St. Mary's Hospital, Imperial College NHS Trust, London W2 6HP, UK

## Abstract

*Introduction*. The results of the recent DECRA study suggest that although craniectomy decreases ICP and ICU length of stay, it is also associated with worst outcomes. Our experience, illustrated by these two striking cases, supports that early decompressive craniectomy may significantly improve the outcome in selected patients. *Case Reports*. The first patient, a 20-year-old man who suffered severe brain contusion and subarachnoid haemorrhage after a fall downstairs, with refractory ICP of 35 mmHg, despite maximal medical therapy, eventually underwent decompressive craniectomy. After 18 days in intensive care, he was discharged for rehabilitation. The second patient, a 23-year-old man was found at the scene of a road accident with a GCS of 3 and fixed, dilated pupils who underwent extensive unilateral decompressive craniectomy for refractory intracranial hypertension. After three weeks of cooling, paralysis, and neuroprotection, he eventually left ICU for rehabilitation. *Outcomes*. Four months after leaving ICU, the first patient abseiled 40 m down the main building of St. Mary's Hospital to raise money for the Trauma Unit. He has returned to part-time work. The second patient, was decannulated less than a month later and made a full cognitive recovery. A year later, with a titanium skull prosthesis, he is back to part-time work and to playing football. *Conclusions*. Despite the conclusions of the DECRA study, our experience of the use of early decompressive craniectomy has been associated with outstanding outcomes. We are currently actively recruiting patients into the RESCUEicp trial and have high hopes that it will clarify the role of the decompressive craniectomy in traumatic brain injury and whether it effectively improves outcomes.

## 1. Introduction and Objectives

There is a considerable controversy [1, 2] regarding whether decompressive craniectomy improves the functional outcome in patients with severe traumatic brain injury and refractory raised intracranial pressure. According to the study recently published by Ma et al. [3], this procedure decreases intracranial pressure and length of stay in the ICU but is associated with more unfavourable outcomes according to the GOSE score. Our perception of this procedure differs from the conclusions drawn by these colleagues.

## 2. Patients and Methods

**Case 1**

**Figure 1:**
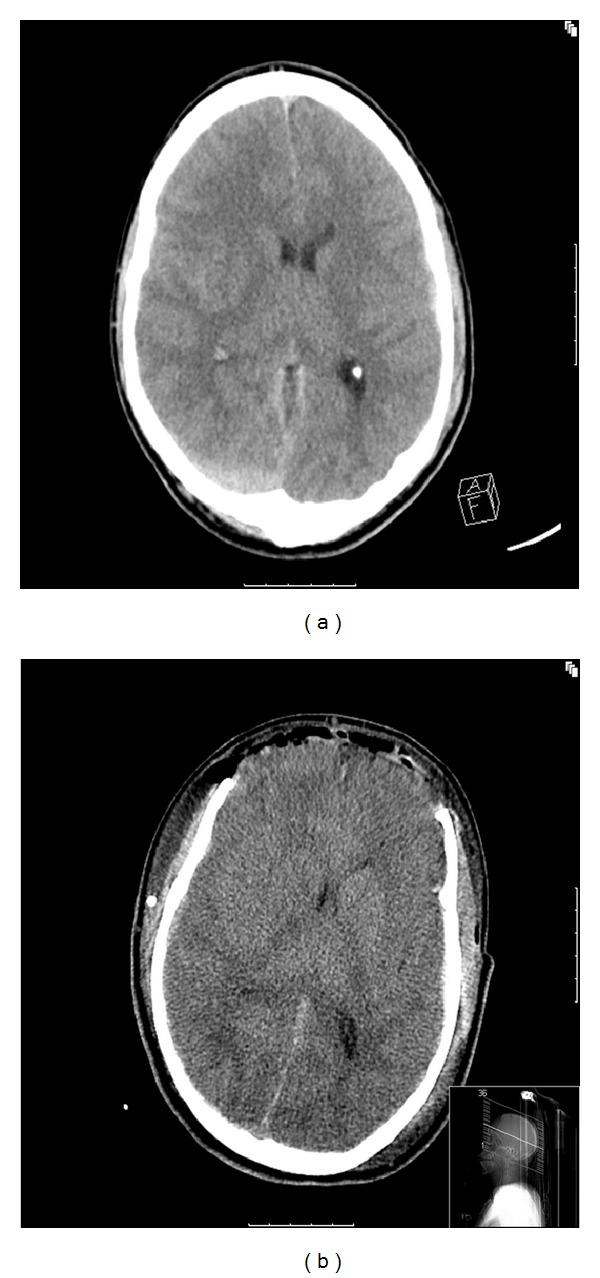


A 23-year-old male was found at the bottom of the stairs with initial GCS 3/15 on scene but recovered to GCS 7/15 before being transferred to a local hospital by LAS, where a CT head was performed (Figure 1(a)) showing bifrontal contusions and subarachnoid blood with evidence of ICP. He was then transferred to St. Mary's Hospital six hours later where he was immediately taken to theatre to undergo an ICP bolt insertion. He was then admitted to our ICU where aggressive medical treatment was commenced despite which the ICP raised up to 35 mmHg. After 2 hours without improvement, he was transferred back to theatre where a bifrontal craniotomy was performed and subdural blood evacuated. Due to refractory ICP and evolving cerebral oedema, he was taken back to theatre 24 hours later to undergo an extensive bifrontal decompressive craniectomy (Figure 1(b)) and the evacuation of a right frontal subdural haematoma, requiring neuroprotection for another 48 hours. The bone flap was left in his abdomen for further reconstruction. An early tracheostomy was performed at day 4 of admission to allow early weaning but complicated by an RLL pneumonia needing deep sedation to control ventilation and ICP. Despite that an EEG did not demonstrate seizures, he was commenced on phenytoin prophylactically.

The ICP bolt was removed 11 days after surgery as the patient was in the process of waking up and had been under control for more than 48 h. 

Agitation and withdrawal to benzodiazepines delayed his weaning, but his agitation was eventually controlled with a clonidine infusion, haloperidol, and chlordiazepoxide.

 A head and neck MRI at day 12 showed mutiple previously known contusions bilaterally. Eventually, he was weaned and decannulated after 18 days in ICU. He was discharged to our major trauma ward.

**Case 2**

A 20-year-old male was found 10 m from a car with both airbags deployed. With an intact windscreen it was assumed that he had self-extracated from the car. GCS was 3/15 on scene with bilateral fixed and dilated pupils. He was attended by London's Air Ambulance that intubated him and performed bilateral thoracostomies. A CT scan at St. Mary's hospital showed a left subdural haemorrhage and extensive cerebral oedema. He was then taken to theatre urgently where he underwent a left frontoparietal craniotomy + evacuation of subdural haemorrhage + insertion of ICP transducer. He had a subgaleal drain left in situ and his ICP at the end of the procedure was 8 mmHg.

**Figure 2:**
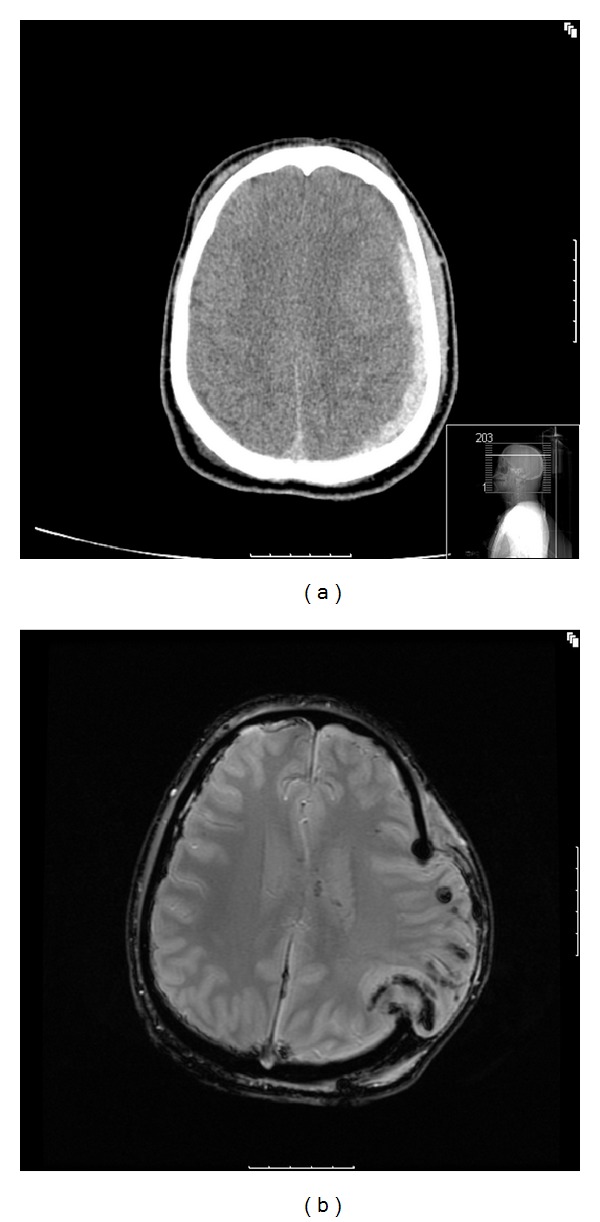


Transferred to ICU postprocedure where aggressive medical therapy was commenced despite which the patient developed intracranial hypertension. After a second CT head, he was taken by the neurosurgeons to theatre to undergo an extensive left-sided decompressive craniectomy (Figure 2(a)). The bone flap was discarded.

The first sedation hold showed flexion posturing to pain stimuli. He was resedated for neuroprotection and cooled down to normothermia.

An MRI brain done at day 4 showed significant brain herniation through craniectomy when lying flat for scan (Figure 2(b)). There is also some evidence of infarction around the area of herniation.

He developed early ventilator-acquired pneumonia needing deep sedation, paralysis, and active cooling to control his ICP. Intracranial hypertension occurred each time paralysis and active cooling were stopped. Therefore, both continued for 3 weeks.

A percutaneous tracheostomy was performed at day 18 when ICP allowed to proceed, and a PEG was inserted at day 19 without complications.

He subsequently developed a significant element of autonomic storming with hypertension, pyrexia, and tachycardia.

He had a hygroma at the opposite site of the surgery evacuated at day 29 via burr holes. Several EEGs showed low amplitude, no seizure activity, and poor prognosis.

EMG at day 33 showed severe critical care myopathy. He had no peripheral neuropathy. Sedation and ventilation gradually were weaned off until he was discharged to our major trauma ward breathing spontaneously via his tracheostomy, definitely tracking and withdrawing to painful stimuli.

## 3. Results

The first young boy is back to part-time work and raised funds for our ICU by abseiling St. Mary's hospital side (130 feet) only 4 months after-ICU stay.

The second young boy, less than a month later, was decannulated and underwent rehabilitation with a GCS of 15/15 being cognitively recovered. A year later, with a titanium skull prosthesis, he is back to part-time work and to playing football.

## 4. Conclusions

In spite of the conclusions of the DECRA study, our experience with the use of the early decompressive craniectomy has shown outstanding outcomes, with these presented cases being probably the most striking. We consider the ICP at which the DECRA patients were randomised very low (20 mmHg); hence probably many people underwent the operation (with its morbidity). Besides, our two patients presented with fixed dilated pupils. Despite being randomized, more patients in the craniectomy arm of the DECRA had unreactive pupils (after randomization but before surgery) than patients in the medical therapy arm, a potential confounding factor. The question always may be to identify suitable patients for a certain therapy. These two cases were very young (20 and 23) and without severe comorbidities. We are currently an active recruiter of the RESCUEicp trial that we hope will help clarify the role of the decompressive craniectomy in TBI and whether it effectively improves outcomes.

## Abbreviations

DECRA study: Early decompressive craniectomy in patients with severe traumatic brain injury
ICU: Intensive care unit
GOSE: Extended Glasgow Outcome Scale
ICP: Intracranial pressure
GCS: Glasgow Coma Scale
RESCUE-ICP: Randomised Evaluation of Surgery with Craniectomy for Uncontrollable Elevation of Intra-Cranial Pressure
LAS: London Ambulance System
RLL: Right lower lobe
EEG: Electroencephalogram
PEG: Percutaneous endoscopic gastrostomy.
